# Changing the paradigm for primary research dissemination

**DOI:** 10.12688/wellcomeopenres.20715.1

**Published:** 2024-02-15

**Authors:** Ashley Farley, Kazuhiro Hayashi, Eva Hnatkova, Hans de Jonge, Heather Joseph, Robert Kiley, Ruth King, Rebecca Lawrence

**Affiliations:** 1 Bill & Melinda Gates Foundation, Seattle, Washington, USA; 2 National Institute of Science and Technology Policy, Tokyo, Japan; 3 National Library of Technology (NTK), University of Chemistry and Technology, Prague, Czech Republic; 4 Dutch Research Council, NWO, The Haque, The Netherlands; 5 SPARC, Washington DC, USA; 6 cOAlition S, Strasbourg, France; 7 Open Research Central, London, UK; 8 F1000 Research Ltd, London, UK

**Keywords:** Open research, scholarly infrastructure, research validation, research dissemination, cross-stakeholder, funding.

## Abstract

The predominant research publishing system is not equitable by design, nor optimised to advance research to create knowledge and ultimately to benefit society. Open Research Central (ORC) was created to foster the re-imagination of the research dissemination system to facilitate trust, transparency and equitable participation. In five years of operation, before dissolving, the non-profit organisation produced outputs and learnings valuable to the development of a responsible research dissemination system. We are sharing our experience in the hope that it will provide others who share the same vision and goals with useful materials to build on. We think that there remains a need for global, cross-stakeholder exploration to build collective understanding of research validation and dissemination and to pilot solutions. However, as this article will explore, enabling and supporting the development of such a collective voice and consequent action is a challenging endeavour in the current landscape and funding environment.

## Disclaimer

The views expressed in this article are those of the authors. Publication in Wellcome Open Research does not imply endorsement by Wellcome.

## Introduction

Open Research Central (ORC) operated as a non-profit organisation governed by a Board of Directors from 2018–2023. The Board was expanded in 2020 to comprise representatives of different stakeholder groups from across the scholarly system, who came together with a shared interest in open research and an ambition to improve the way research findings are validated and disseminated.

**Figure 1.**
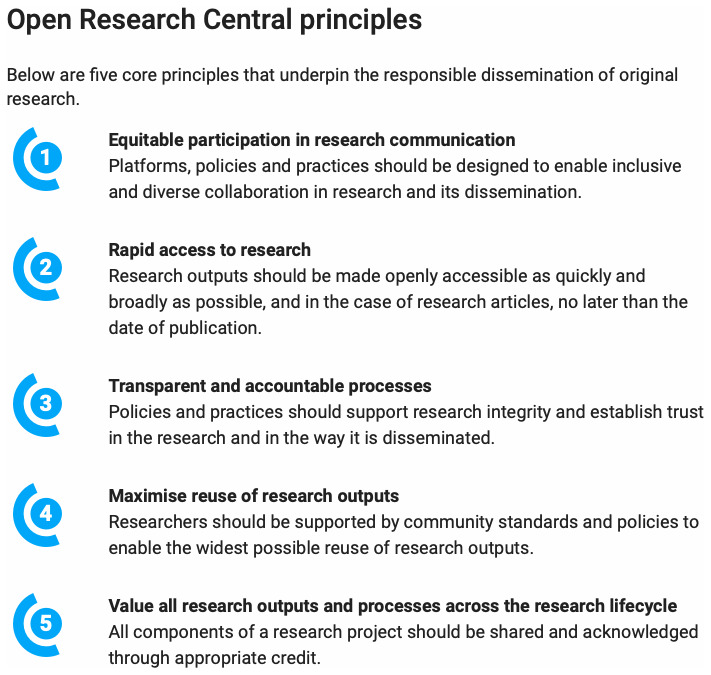
Open Research Central principles.

The ORC vision was to contribute to creating a world in which research outputs are openly disseminated and responsibly evaluated to maximise their benefits to society. To foster the re-imagination of the research dissemination system to facilitate trust, collaboration, and transparency, the Board created a set of five core principles that underpin the responsible dissemination of original research ( Figure 1). These principles provided a critical foundation and framework for ORC and can be freely used and built on by others working in this area.

**Figure 2.**
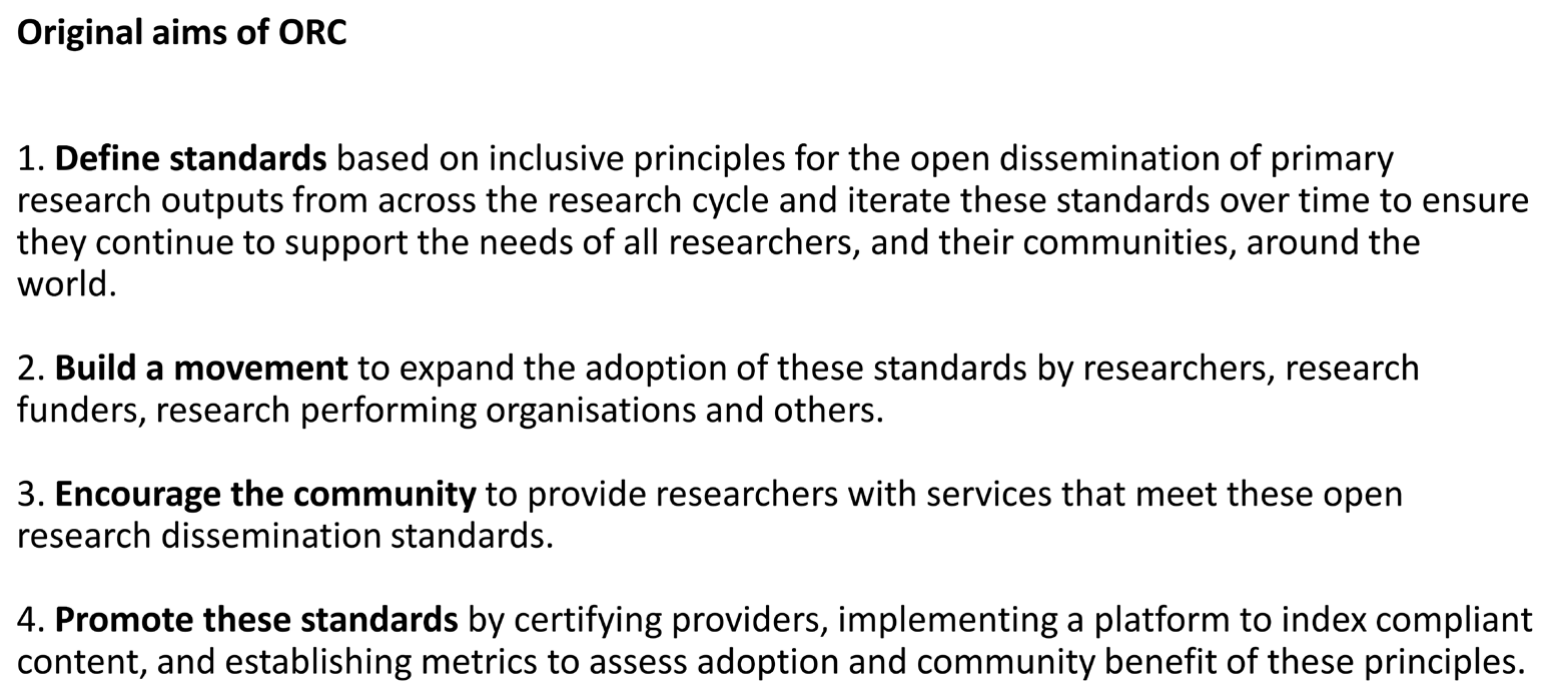
Original aims of ORC.

ORC’s aim was to build the movement by advocating for an increase in adoption of the principles *via* open practices, such as post-publication transparent peer review, inclusion of data and code availability statements in articles and dissemination of all components of a research project. The original plan was to validate those practices through certification of compliant content and to make the content visible through indexing on an ORC platform. At the same time, developing a community of members to identify and overcome barriers to responsible research dissemination ( Figure 2).

ORC received funding from Wellcome and F1000 Research Ltd to employ a part-time Programme Director for an 18-month period. The role of the Programme Director was to support the full range of activities required for ORC to achieve its mission. Small organisations face a high burden of organisational administration just to exist and to employ someone. F1000 made this phase feasible by employing and hosting the Programme Director on behalf of ORC and by providing and maintaining the website gratis. The intention of the ORC Board was that after an initial phase to achieve sustainability, these functions would transfer fully to ORC.

In 2023, after deep consideration of different options, the Board concluded that the set-up of ORC wasn’t right for the goals and took the decision to dissolve. Here, we describe the challenges that led to that decision along with our positive experiences of working on ORC for the benefit of other organisations working in this space. We also make a case for subsequent work that we believe is critical to achieving responsible research dissemination systems. 

## Fast-evolving landscape

During the period that ORC operated, there was significant growth in new initiatives, with policy developments and an expansion of the options available to researchers to openly publish their research outputs. 

Specifically, we have witnessed unprecedented international consensus on the benefits of open science with the release and ratification of the UNESCO Recommendation on Open Science ^1^. We’ve also seen movement towards increased transparency in publishing processes ^2, 3^, a rise in the use of preprints ^4^ and new models separating the validation of research from dissemination ( *e.g.* PREreview, Peer Community In and Review Commons).

The conversation has moved beyond open access and open research to the wider challenges in creating responsible research dissemination systems. Building on the foundation laid by the San Francisco Declaration on Research Assessment ( DORA), we now have active efforts to reform research assessment in Europe ( CoARA), the United States ( HELIOS), and Latin America ( FOLEC - CLACSO). Equitable participation in knowledge sharing has been brought squarely into focus, tackling barriers such as article-based charges to publish ^5, 6^.

Along with this flurry of positive activity, however, we have experienced instances of polarity and mistrust. High-profile cases reported by publishers have uncovered that the processes designed to uphold research integrity are being manipulated on a wide scale through papermills, peer review rings and other malpractice ^7^. Community members have come together to tackle the problem ^8^ but the crisis in research integrity has exacerbated a long-standing distrust of the power and motivations of commercial publishers. At the same time, calls by the EU for its members to create nonprofit open access models for publication of research involving public funds have fuelled tension and uncertainty about working relationships between public organisations and commercial publishers ^9^.

## Revenue models for transformational change

To drive uptake in adoption of the ORC principles by both providers and the researcher community, one of ORC’s original goals was to certify content that met the ORC principles and to index it on a platform. When we derived criteria from the principles to assess a group of providers of innovative journals and platforms, there were very few offering a model that would adhere to all the requirements. Part of the thinking around financial sustainability was to build a membership revenue model from those whose content was indexed in the platform but it soon became clear that not enough venues were eligible to build such a model. Those that were eligible were typically from smaller start-ups or relatively unfunded groups.

We debated the balance between reducing the bar to include more of the community, risking making less progress and differentiating ourselves less from other activities, *versus* adopting a position that really pushed for more transformational change to try to accelerate progress but struggling for sustainability. Ultimately, with equity as a core principle, we didn’t want to create a membership structure that would exclude many groups.

We also recognised that inclusion criteria based on specific elements of open research was too reductive and risked entrenching inequalities. We needed to support a diverse publication landscape that met the needs of different scholarly communities.

Alternative mechanisms that we explored came up against the same challenge of finding a sustainable revenue model for emergent work with a small core community. For example, we considered developing a collective of the pioneers in responsible research dissemination. Under this approach we would provide a space for the innovators breaking boundaries in research validation and dissemination and for the infrastructure providers, funders and institutions actively wanting to drive a shift.

The innovators told us they needed amplification of voice; validation; cross-stakeholder trust-building; help to contend with infrastructure such as indexing not designed for their different methods of versioning and transparent peer review; support to become sustainable. They were confronted with a huge industry, with entrenched rules and norms. They needed outside support to create an environment where new systems that put the focus on open and equitable access to research outputs could flourish.

Sustainable revenue models remain challenging even for successful and embedded open scholarly infrastructure ^10^. The DOAJ carried out a sustainability review which demonstrated that open scholarly infrastructures need to carve out distinct value propositions that resonate with different communities in order to maximise opportunities for external support. Revenue implicitly can’t be made from content and a mix of mechanisms is often pursued with organisations operating on stretched budgets ^11^.

## Grant support for transformational change

Ultimately, the aim of ORC to support emergent practices and organisations was not revenue-generating. F1000 provided short-term funding and hosting support. However, in an environment of increased sensitivity to the roles of commercial publishers, it raised questions from the community about the perceived independence of the organisation from F1000 and concerns that it would hamper wider support and engagement.

Alternative grant opportunities were challenging, which is often the case for early-stage organisations where funding is required for exploratory work ahead of having more concrete outcomes. ORC’s model didn’t fit with the strategy or focus areas of many funders and it was challenging to make a strong case without being able to demonstrate a clear value proposition. Grant application processes often request a route to sustainability. For transformational change, that requires you to say how the work will thrive in the prevailing system, while simultaneously working to change that system; it’s not easy. Grant funding cycles can be more than one-year long, which was hard for ORC to join up with the initial period of support.

## Recommendations for progress in responsible research dissemination

**Figure 3.**
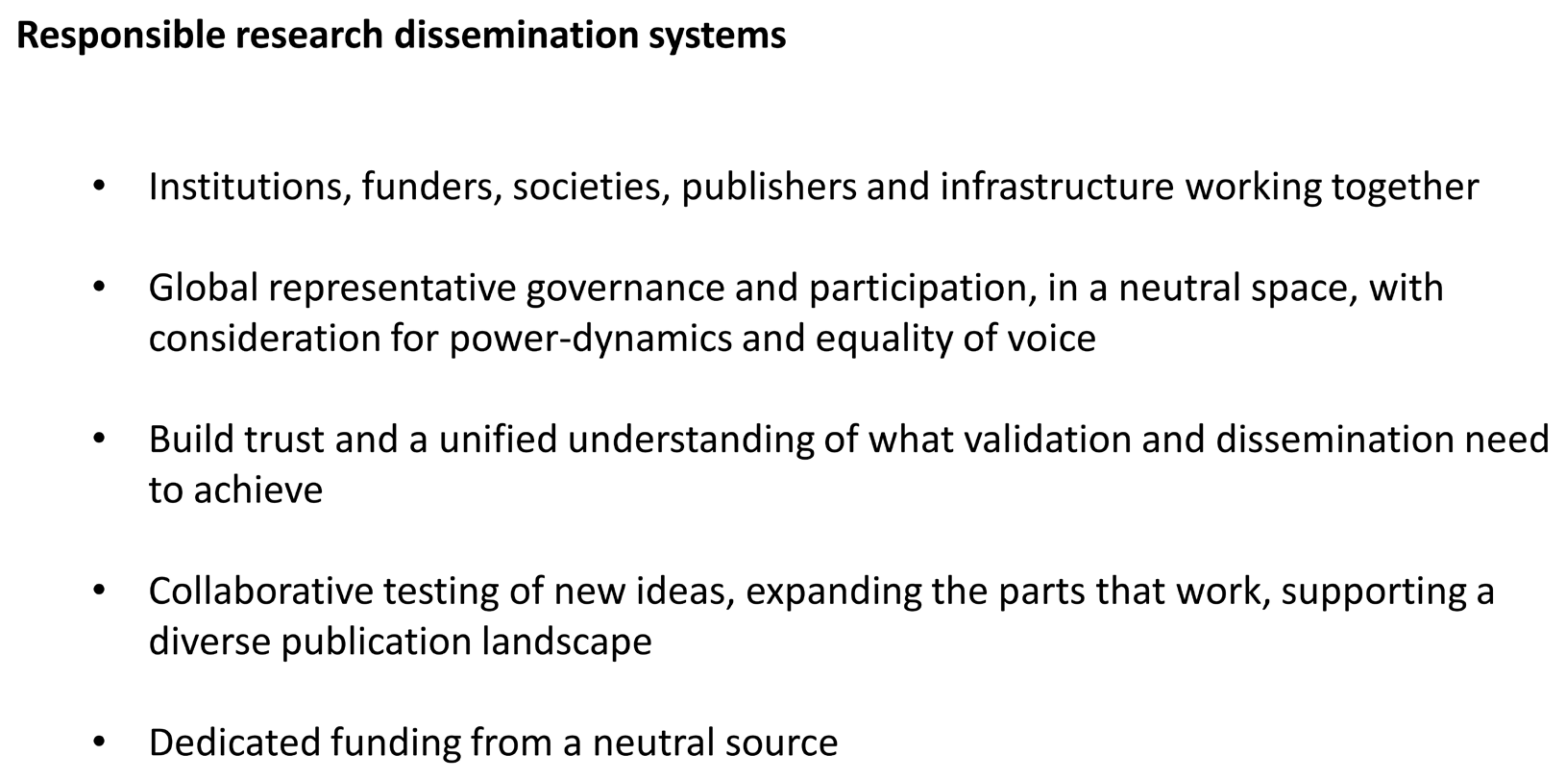
Responsible research dissemination systems.

The experiences of ORC have helped us identify the piece we believe is missing for sustainable progress in responsible research dissemination. Specifically, responsible research dissemination systems will only be created when institutions, funders, societies, publishers, infrastructure service providers and the researcher community work together. It requires global representative governance and participation, in a neutral space, with consideration for power dynamics and equality of voice. The aim is a common understanding of what validation and dissemination need to achieve, leading to suggested solutions and real tangible action in testing those solutions in the real world. It all needs to be securely funded and adequately resourced ( Figure 3).

As part of this work, we need to hold space for trust to be built between stakeholders. They need to understand each other’s different values, motives and challenges, with each group acknowledging the barriers they currently create. Participants need to be willing to step into a challenging collaborative process and be invested in finding solutions.

ORC demonstrated the value of holding space for detailed conversation between stakeholders. We made great progress and it took time to develop that valuable thought-partnership amongst the members of the Board. With the relationship rooted in practical work ( *e.g.* developing the principles) the members combined their different perspectives from funders, governmental organisations, infrastructures, publishers and researchers to achieve shared understanding and new outlooks.

The value was also demonstrated by the Open Science Policy Platform ^12^, which ran from 2016 to 2020. A collection of the major stakeholders was convened to advise the European Commission on how to develop its Open Science Policy. The different parts of the system were represented around the table at the same level, which enabled discussion about some of the most challenging issues. Although everyone was supportive in principle about the benefits of open research, it was clear that it meant different things to different stakeholders and there were challenges which were not recognised or understood between each other.

A pragmatic approach to finding solutions is needed, given the complexity of the research ecosystem with its many actors, global differences and embedded cultures. The work needs to take account of related issues in the wider research ecosystem, such as research assessment reform, without trying to solve everything. These are example questions to begin to address:

- What are the principles of research validation and dissemination? What do those processes need to achieve and how do we need to work together across the community to deliver that most effectively?
- How do research validation and dissemination interact with the wider research ecosystem? For example, how do cultures of hierarchy and prestige in research interact with validation and dissemination? How does global research collaboration interact with funding, institutional and research infrastructures that are organised at national level?
- Is there a tension between standardisation and inclusivity? How do we prevent standards from entrenching inequalities?

Finally, the process needs to result in action. There is a powerful opportunity for collaborative testing of new ideas, moving beyond discussion to achieve real progress. We need willing parties to come together and pilot new approaches, then expand the parts that work and reflect on the ones that don’t. This segment needs to take account of how challenging the environment is for initiatives that go against the status quo, providing the pilots with the stability and support they need to have a chance of succeeding.

## Conclusions

The mission of ORC to re-imagine the research dissemination system remains relevant. The current landscape brings many challenges and inequalities to enabling research to address global challenges at the rate required by society. Activity in this space is increasingly cross-stakeholder and moving beyond open research to focus on the most important challenges including equitable participation. However, there is a continuing risk that stakeholders may move further apart in an environment of uncertainty and mistrust. 

ORC’s experience of attempting to envision a collaborative, sustainable mechanism, while living its values, can be seen as an opportunity to learn. Securing a sustainable revenue stream was certainly impacted by the challenge of finding a clear and distinct value proposition that resonated with a large-enough group, in a fast-paced environment. But ultimately, the aim of ORC to support emergent practices and organisations simply wasn’t revenue-generating. The grant funding landscape is extremely competitive and hard to navigate without a plan for sustainability. The provision of resources from commercial publishers to support this work currently brings motives and neutrality into question.

Responsible research dissemination systems will only be created when institutions, funders, societies, publishers, infrastructures and the researcher community work together. It requires global representative governance and participation, in a neutral space, with consideration for power-dynamics and secure funding. We need to hold space and time for trust to be built between stakeholders, aiming for a unified understanding of what validation and dissemination need to achieve. That unified understanding is a precursor for the right systems and the right pilot solutions to emerge. The work needs to be pragmatic, given the complexity of the landscape, and pilot solutions need to be adequately supported.

## Data Availability

No data are associated with this article.

## References

[1] UNESCO Recommendation on Open Science.2021; Accessed 3 November, 2023. Reference Source

[2] Research Data Alliance. Accessed 3 November, 2023. Reference Source

[3] The Future of Peer Review is Open. Research Professional News. Accessed 3 November, 2023. Reference Source

[4] Puebla I Polka J Riega OY : Preprints: Their Evolving Role in Science Communication. *MetaArXiv.* 2021. 10.31222/osf.io/ezfsk

[5] Beyond article-based charges: working group established. Plan S,2023; Accessed 9 November 2023. Reference Source

[6] Working to create equity in open access.2023; Accessed 9 November 2023. Reference Source

[7] Candal-Pedreira C Ross JS Ruano-Ravina A : Retracted papers originating from paper mills: cross sectional study. *BMJ.* 2022;379: e071517. 10.1136/bmj-2022-071517 36442874 PMC9703783

[8] Research Integrity - STM.2022; Accessed 9 November 2023. Reference Source

[9] Council Conclusions on High-Quality, Transparent, Open, Trustworthy and Equitable Scholarly Publishing.2023; Accessed 9 November 2023. Reference Source

[10] Who’s afraid of open infrastructures? Research Information.2023; Accessed 9 November 2023. Reference Source

[11] Striking a balance between openness and free access in scholarly infrastructure - DOAJ at 20 LSE Impact Blog.2023; Accessed 9 November 2023. Reference Source

[12] Open Science Policy Platform: Final report.2020; Accessed 9 November 2023. Reference Source

