# Supplementary material for: Changing the paradigm for primary research dissemination
Source: Wellcome Open Res. 2024 Feb 15;9:37. [Version 1] doi: 10.12688/wellcomeopenres.20715.1 (PMC11391192; doi:10.12688/wellcomeopenres.20715.1)
Supplement: Peer review history

# Reviewer report: Reviewer response for version 1

**DOI:** 10.21956/wellcomeopenres.22924.r96073

**Published:** 2024-09-12

**Authors:** Morufu Olalekan Raimi

**Affiliations:** 1 0Department of Environmental Management and Toxicology, Faculty of Sciences, Federal University Otuoke, Bayelsa State, Nigeria

The theme of your open letter titled *"Changing the Paradigm for Primary Research Dissemination"* is indeed important and especially for contexts of Research dissemination, this theme should indeed receive greater attention and to that end I would like to thank you for bringing together this writeup on this issue.

**However, the paper is challenged on many fronts with regard to the implementation of this larger objective:**

1. The article title can be recasted to reflect ORC. For example, “Lessons from Open Research Central: Changing the Paradigm for Primary Research Dissemination” This would give potential readers a clearer understanding of the content and its unique focus, particularly for search engines and citation contexts. Hence, it would make the subject matter clearer and attract the right audience.
2. The abstract accurately reflects the content, **but **Abstract should lay out five key points:
**Rationale** (1-2 sentences) - why was the research needed?
**Objective **(1 sentence)- what were you trying to provide to meet that need
**Method**(s) (up to 3 sentences) - briefly summarize what and which parameters were measured.
**Results** (up to 4-5 sentences)- what did you find? Please add some data to demonstrate the findings.
**Conclusions/Recommendations** (1 sentence) - so what should be done with or in response to your findings?
3. Keywords are appropriate.
4. Novelty of the work is established but could be improved.
5. The manuscript is generally well-written, but simplifying some of the more technical terms would make it accessible to a broader audience, including those who may not be as familiar with scholarly publishing trends.
6. Broaden the scope of the manuscript by addressing how the insights gained from ORC can apply to a wider range of research dissemination models, including those not primarily focused on open access. A discussion on how other fields could benefit from these lessons would enhance its relevance.
7. The manuscript acknowledges that ORC dissolved but does not provide a deep reflection on the specific reasons for its failure. It lacks critical analysis of what exactly went wrong and what could have been done differently.
8. The discussion on sustainability needs to be expanded, the discussion on alternative funding strategies and sustainability models need to be emphasized upon. Consider providing examples of successful open access initiatives that have overcome similar challenges. This could help other organizations facing similar issues.
9. Expand on the global context of research dissemination, addressing how initiatives like ORC could navigate diverse research environments. Discuss the challenges and opportunities for global partnerships, particularly in regions with underdeveloped research infrastructures.
10. Provide more emphasis on the innovative approaches ORC attempted or could have explored. Detailing novel methods of research dissemination that were tested (e.g., open peer review models, data and code sharing practices) would offer readers a more comprehensive understanding of what was attempted and what could be improved.
11. The recommendations need to be improved upon as it lacks specificity and actionable insights. While it highlights important issues, it doesn’t offer concrete, step-by-step guidance for other organizations that might attempt similar initiatives.
12. Ensure all citations are thoroughly checked for accuracy. While the referencing is robust, ensure that they are current and fully integrated with the discussion points.
13. There is enough new content in this paper to distinguish it from other works.
14. The submission provides enough new material for journal indexing.

This manuscript presents an important reflection on the challenges and opportunities in transforming research dissemination. By addressing the points above, particularly focusing on specificity in recommendations and expanding the discussion on sustainability, it will be even more impactful.

Does the article adequately reference differing views and opinions?

Yes

Are all factual statements correct, and are statements and arguments made adequately supported by citations?

Yes

Is the Open Letter written in accessible language?

Yes

Where applicable, are recommendations and next steps explained clearly for others to follow?

Partly

Is the rationale for the Open Letter provided in sufficient detail?

Yes

Reviewer Expertise:

Biostatistics and Research Methodology, Environmental Epidemiology, Health Economics, Pollution Control Management, Hydrogeochemistry, Groundwater Pollution, Groundwater Quality, Health Risk Assessment, Water Pollution and Management, Water and Gender, Chemicals & Health, Environmental Public Health Policy & Practice, Environmental Justice & Health Equity, Noise & Health, Carbon Accounting Services, Environmental & Health Impact Assessments, Community based participatory Research, Climate Change Mitigation and Adaptation, Oil Spills Clean up and Remediation, Environmental Toxicology & Health, Emissions Control and Respiratory Protection.

I confirm that I have read this submission and believe that I have an appropriate level of expertise to confirm that it is of an acceptable scientific standard, however I have significant reservations, as outlined above.

# Reviewer report: Reviewer response for version 1

**DOI:** 10.21956/wellcomeopenres.22924.r96075

**Published:** 2024-09-11

**Authors:** Roslina Othman

**Affiliations:** 1 Kulliyyah of Information and Communication Technology, International Islamic University, Kuala Lumpur, Malaysia

This paper discusses five core principles to foster trust, collaboration, and transparency in the research dissemination system, providing a foundation for future research. ORC aimed to promote responsible research dissemination through open practices like transparent peer review and data availability statements. 

 However, in 2023, the ORC Board decided to dissolve due to its unsuitable setup for its goals. Research integrity is being manipulated through malpractice and papermills, leading to polarity and mistrust among community members.  Sustainable revenue models remain challenging even for successful and embedded open scholarly infrastructure. Responsible research dissemination systems will only be created when institutions, funders, societies, publishers, infrastructure service providers and the researcher community work together. 

 There is a need for willing parties to collaborate and pilot new techniques, then expand on what works and reflect on what doesn't. Responsible research dissemination systems will only be created when institutions, funders, societies, publishers, infrastructures and the researcher community work together. 

 Does the article adequately reference differing views and opinions? Partly. The paper does not mention the terms "open humanities" and "open scholarship," even though it mentions their concepts.

 Where applicable, are recommendations and next steps explained clearly for others to follow? Partly.

 "The work needs to be pragmatic" needs to be elaborated.

Does the article adequately reference differing views and opinions?

Partly

Are all factual statements correct, and are statements and arguments made adequately supported by citations?

Yes

Is the Open Letter written in accessible language?

Yes

Where applicable, are recommendations and next steps explained clearly for others to follow?

Partly

Is the rationale for the Open Letter provided in sufficient detail?

Yes

Reviewer Expertise:

Information retrieval, bibliometrics, open science, knowledge management, future studies

I confirm that I have read this submission and believe that I have an appropriate level of expertise to confirm that it is of an acceptable scientific standard.

## References

[1] : Toward Open Research: A Narrative Review of the Challenges and Opportunities for Open Humanities. *Journal of Communication* .2021; 10.1093/joc/jqab028 10.1093/joc/jqab028

# Reviewer report: Reviewer response for version 1

**DOI:** 10.21956/wellcomeopenres.22924.r96070

**Published:** 2024-09-11

**Authors:** Moumita Koley

**Affiliations:** 1 DST-Centre for Policy Research, Indian Institute of Science, Bangalore, India

This open letter offers valuable insights into the Open Research Central (ORC) initiative, which aimed to reimagine scholarly communication by fostering trust, transparency, and equitable participation in the system. It also shows why ORC ultimately could not fulfill its promise and had to be dissolved.

 However, the principles outlined by ORC remain highly relevant, emphasizing the need for such values to be adhered to in order to build a better scientific communication system. Organizations like the International Science Council and Plan S also endorse similar principles. On the operational side, the original aims of ORC were difficult to achieve, and apart from many other reasons, the revenue-generation part was difficult.  

 The letter gives us a compelling example of why a more collaborative approach among all stakeholders is essential for driving meaningful change in scientific publishing. With numerous new initiatives emerging, synergy is crucial to ensure their sustainability and to present a value-added proposition for the research community. This will help convince researchers that the current inequitable and inefficient system needs to be replaced.

 Overall, the letter adds value for all stakeholders advocating for a better system.

Does the article adequately reference differing views and opinions?

Partly

Are all factual statements correct, and are statements and arguments made adequately supported by citations?

Yes

Is the Open Letter written in accessible language?

Yes

Where applicable, are recommendations and next steps explained clearly for others to follow?

Yes

Is the rationale for the Open Letter provided in sufficient detail?

Yes

Reviewer Expertise:

Science policy, open science, research evaluation

I confirm that I have read this submission and believe that I have an appropriate level of expertise to confirm that it is of an acceptable scientific standard.

## References

[1] : A combination model of CT-based radiomics and clinical biomarkers for staging liver fibrosis in the patients with chronic liver disease. *Sci Rep* .2024;14(1) : 10.1038/s41598-024-70891-9 20230 10.1038/s41598-024-70891-9 39215041 PMC11364870

# Reviewer report: Reviewer response for version 1

**DOI:** 10.21956/wellcomeopenres.22924.r88182

**Published:** 2024-07-30

**Authors:** Mikael Laakso

**Affiliations:** 1 Hanken School of Economics, Helsinki, Finland

This letter is anchored in a post-mortem reflection of the founding, operation, and dissolution of Open Research Central (ORC).

 The ORC was a non-profit organisation that was operational between 2018-2023 and had ambitious goals of pioneering and promoting progressive open practices for open research dissemination. It is well known that the landscape of scholarly communication is currently undergoing a lot of change, offering new opportunities for research dissemination, and the letter does a good job at describing how the ORC was designed to be aligned and support many of these emerging practices. In the letter the perspective is primarily looking from the inside out, giving valuable insight to the decisions taken and the challenges faced as time went on, both inside and outside the organisation.

 The letter is well-written, easy to follow and understand, and integrates a useful set of external references into its description that broadens up the perspective from a narrow narrative into something that is woven into the broader context of different types of change happening in the research dissemination landscape. It includes a balanced description of strategic goals, organizational/administrative aspects, and a very meaningful discussion on sustainability/funding which I am sure many other initiatives in this space will be able to relate to. A strength of the letter is how it sheds light on the path forward for work and practices in this area, which is both inspiring and informative. I also think the discussion about balancing the strictness of requirements for eligible members/outputs for progressive initiatives that are intended to scale and grow equitably is also valuable.

 My only suggestion for the authors to optionally reconsider if making a revision is the title, as I think having the ORC mentioned within it would make it more directly describe what the the text is actually about and guide interested readers to it. This would improve its function e.g.  within reference lists of future article citing it, or search engines that do not display the full abstract, to see more clearly what the text is about. The focus is of course broader than just the ORC but I think that is still this letters strongest unique part and could be highlighted more.

 I think it is a great idea that lessons learned from initiatives like this are made public so that they can fuel new iterations and ideas that continue on from where work once ended.

Does the article adequately reference differing views and opinions?

Yes

Are all factual statements correct, and are statements and arguments made adequately supported by citations?

Yes

Is the Open Letter written in accessible language?

Yes

Where applicable, are recommendations and next steps explained clearly for others to follow?

Yes

Is the rationale for the Open Letter provided in sufficient detail?

Yes

Reviewer Expertise:

Scholarly communication, Open science, Publishing, Scientometrics, Meta-research

I confirm that I have read this submission and believe that I have an appropriate level of expertise to confirm that it is of an acceptable scientific standard.
